# *XAB2* tagSNPs contribute to non-small cell lung cancer susceptibility in Chinese population

**DOI:** 10.1186/s12885-015-1567-4

**Published:** 2015-07-31

**Authors:** Na Pei, Lei Cao, Yingwen Liu, Jing Wu, Qinqin Song, Zhi Zhang, Juxiang Yuan, Xuemei Zhang

**Affiliations:** 1 Institute of Molecular Genetics, College of Life Sciences, Hebei United University, Tangshan, 063000 China; 2 Department of Epidemiology, College of Public Health, Hebei United University, Tangshan, 063000 China; 3 Tangshan Gongren Hospital, Hebei United University, Tangshan, China

**Keywords:** *XAB2*, Lung cancer, Polymorphisms, Transcriptional coupling nucleotide excision repair, Susceptibility

## Abstract

**Background:**

XPA-binding protein 2 (XAB2) interacts with Cockayne syndrome complementation group A (CSA), group B (CSB) and RNA polymerase II to initiate nucleotide excision repair. This study aims to evaluate the association of *XAB2* genetic variants with the risk of non-small cell lung cancer (NSCLC) using a tagging approach.

**Methods:**

A hospital-based case-control study was conducted in 470 patients with NSCLC and 470 controls in Chinese population. Totally, 5 tag single nucleotide polymorphisms (SNPs) in *XAB2* gene were selected by Haploview software using Hapmap database. Genotyping was performed using iPlex Gold Genotyping Asssy and Sequenom MassArray. Unconditional logistic regression was conducted to estimate odd ratios (ORs) and 95 % confidence intervals (95 % CI).

**Results:**

Unconditional logistic regression analysis showed that the *XAB2* genotype with rs794078 AA or at least one rs4134816 C allele were associated with the decreased risk of NSCLC with OR (95 % CI) of 0.12 (0.03–0.54) and 0.46 (0.26–0.84). When stratified by gender, we found that the subjects carrying rs4134816 CC or CT genotype had a decreased risk for developing NSCLC among males with OR (95 % CI) of 0.39 (0.18–0.82), but not among females. In age stratification analysis, we found that younger subjects (age ≤ 60) with at least one C allele had a decreased risk of NSCLC with OR (95 % CI) of 0.35 (0.17–0.74), but older subjects didn’t. We didn’t find that XAB2 4134816 C > T variant effect on the risk of NSCLC when stratified by smoking status. The environmental factors, such as age, sex and smoking had no effect on the risk of NSCLC related to XAB2 genotypes at other polymorphic sites.

**Conclusions:**

The *XAB2* tagSNPs (rs794078 and rs4134816) were significantly associated with the risk of NSCLC in Chinese population, which supports the *XAB2* plays a significant role in the development of NSCLC.

## Background

Worldwide, lung cancer harbored the highest incidence and mortality rates among all malignant cancers [1, 2]. Non-small cell lung cancer (NSCLC), as the most common type of lung cancer, accounts for 75–80 % of all lung cancer cases [3]. The development of lung cancer was greatly affected by the environmental factors, such as cigarette smoking, alcohol drinking and air pollutants [4–6]. However, evidence has showed that the genetic variants of cancer-related genes are associated with lung risk, which the important role of genetic factors in the development of lung cancer [7–9].

Nucleotide excision repair (NER) is the major DNA repair pathway to remove bulky DNA lesions induced by UV light and environmental carcinogens [10]. NER has two subpathways, global genome NER (GG-NER) and transcription coupling NER (TC-NER). TC-NER is involved in a rapid removal of the damages on the transcribed strands of active genes and a resumption of transcription [11–13]. TC-NER is initiated by arresting RNA polymerase II at DNA lesion site on transcript strand. In the initiation of transcription coupling repair, the TC-NER specific proteins Cockayne syndrome complementation group A (CSA) and group B (CSB) are thought to play an important role in removing the stalled RNA polymorase II and recruiting other DNA repair proteins [14]. Many studies have demonstrated that the decreased expression of CSA and CSB in lung cancer and the genetic variants in these two genes were associated with the lung cancer risk [15–18].

Xeroderma pigmentosum group A (XPA)-binding protein 2 (XAB2), which located at 19p13.2, was first identified as an interacting protein with XPA and hence found to interact with CSA, CSB and RNA polymerase II to participant to TC-NER and transcription [17, 19, 20]. *In vitro*, when cells treated with DNA-damaging agents, enhanced interaction of XAB2 with RNA polymerase II or XPA was observed, which suggesting DNA damage-responsive activity of the XAB2 [19].

Due to the important role of *XAB2* in the TC-NER, we proposed that the genetic variants in *XAB2* genes might contribute to the risk of lung cancer. To verify this proposal, we conducted this case-control study to evaluate the role of *XAB2* tagSNPs in the development of NSCLC.

## Methods

### Study population

The study population has been described previously [21]. Briefly, this hospital-based case-control study consisted of 470 patients with NSCLC and 470 cancer-free controls. All subjects were unrelated Han Chinese. All patients with newly diagnosed, and previously untreated primary lung cancer were recruited between January 2008 and December 2012 at Tangshan Gongren Hospital (Tangshan, China). The exclusive criteria included previous cancer and previous radiotherapy or chemotherapy. The controls were randomly selected from cancer-free individuals living in the same region during the same period as the cases were collected. The selection criteria included no prior history of cancer. Controls were frequency matched to the cases by age (±5 years) and sex. At recruitment, informed consent was obtained from each subject who was then interviewed for detailed information on demographic characteristics and lifetime history of tobacco use. The study was approved by Ethics Committee of Hebei United University (Approval No. 12–002).

### Tag SNPs selection and genotyping

Based on the Han Chinese in Beijing (CHB) population data from HapMap database, we used Haploview 4.2 program to select candidate tag SNPs with an r^2^ threshold of 0.80 and minor allele frequency (MAF) greater than 1 %. For XAB2 gene, we extended the 5′- and 3′-untranslated regions (UTR) to include the 5′-UTR and 3′-UTR most SNP. As a result, 5 tagSNPs (2 in 5′ UTR, 2 in intron, 1 in exon region) in *XAB2* were included, which represent the common genetic variants in Chinese population. Genotyping was performed at Bomiao Tech (Beijing, China) using iPlex Gold Genotyping Asssy and Sequenom MassArray (Sequenom, San Diego, CA, USA). Sequenom’s MassArray Designer was used to design PCR and extension primers for each SNP. The information on assay conditions and the primers are available upon request. Genotyping quality control consisted of no-temple control samples for allele peaks and verifying consistencies in genotype calls of 2 % randomly selected duplicate sample. In addition, we excluded individuals and SNPs based on genotyping quality (<90 % call rate).

### Statistical analysis

The *χ*2 test was used to examine differences in demographic variables and the distribution of genotype between patients and controls. Hardy-Weinberg equilibrium (HWE) for each SNP in controls was examined using Pearson goodness-of-fit *χ*2 test. The association of each tag SNP with the risk of NSCLC was estimated by odds ratios (ORs) and 95 % confidential intervals (95 % CI) using unconditional logistic regression adjusted by sex, age, and smoking status. Smokers were considered current smokers if they smoked up to 1 year before the date of cancer diagnosis for NSCLC patients or before the date of the interview for controls. The number of pack-years smoked was determined as an indication of cumulative cigarette-dose level [pack-year = (cigarettes per day/20) × (years smoked)]. Light and heavy smokers were categorized by using the 50th percentile pack-year value of the controls as the cut points (i.e., ≤25 and >25 pack-years). Statistical analysis was performed using the SPSS version16.0 (SPSS Inc, Chicago, IL). A *P* value of < 0.05 was considered as statistically significant. Gene-smoking interaction was analyzed by GxEscan (http://biostats.usc.edu/software).

## Results

### Subject characteristics

The demographic characteristics of all participants are presented in Table 1. The distribution of gender and age among NSCLC cancer cases and healthy controls were not significantly different (*P* = 0.832 for gender, and *P* = 0.470 for age). There were also no significant differences in the distribution of smoking status between cases and controls. However, the heavy smokers (≥25 pack-year) accounted for 63.4 % in cases and only 49.2 % in controls, which suggested that cigarette smoking was a prominent contributor to the risk of lung cancer. Among ever-smokers, 46,8 % (96) and 41.8 % (79) are former smokers in lung cancer cases and controls, respectively. Of 470 NSCLC patients, 37.9 % (178) were adenocarcinoma, 50.6 % (238) was squamous cell carcinoma, and 11.5 % (54) were other types, including large cell carcinoma (*n* = 49) and mixed cell carcinoma (*n* = 5).

**Table 1:** Distributions of select characteristics in cases and control subjects

Variables	Cases (*n* = 470)		Controls (*n* = 470)		*P* value^a^
	No	(%)	No	(%)	
Gender					0.832
Male	324	68.9	328	69.8	
Female	146	31.1	142	30.2	
Age					0.470
<50	84	17.9	96	20.4	
50–59	177	37.7	187	39.8	
60–69	129	27.4	111	23.6	
≥70	80	17.0	76	16.2	
Smoking status					0.321
Non-smoker	265	56.4	281	59.8	
Ever-smoker	205	43.6	189	40.2	
Pack-year smoked					0.001
<25	75	36.6	96	50.8	
≥25	130	63.4	93	49.2	

^a^two-side *χ*2 test

### Selected SNPs and risk of developing NSCLC

The position and minor allele frequency (MAF) of the 5 selected tag SNPs in *XAB2* gene were presented in Table 2. For all selected SNPs, the distributions of genotype frequencies in controls were close to those expected under Hardy Weinberg Equilibrium (HWE) (*P* > 0.05 for all).

**Table 2:** Primary information of tag SNPs inXAB2gene

Gene and locus	Rs number	Contig position	Location	Base change	MAF in controls	*P* for HWE test	Call rate (%)
*XAB2*19p13.2	rs4134816	297747	5′ near gene	T/C	0.04	0.998	100
	rs4134819	297227	5′ near gene	A/G	0.50	0.708	99.8
	rs794083	295863	Intron	C/G	0.33	0.157	100
	rs4134860	290403	Intron	T/C	0.15	0.978	100
	rs794078	289839	T620T	G/A	0.12	0.136	97.8

The observed genotype frequencies in participants and the association of genotypes with the NSCLC were presented in Table 3. Of all selected SNPs in *XAB2* genes, two SNPs were identified to be associated with the risk of NSCLC. For *XAB2* rs794078 G > A polymorphism, we found that AA genotype carriers had a significantly decreased risk for developing NSCLC (OR = 0.12; 95 % CI = 0.03–0.54) in comparison to those with GG genotype. For *XAB2* rs4134816 T > C polymorphism, just one CC genotype was found among all individuals, so we combined CT with CC genotype together for further analysis. Our data showed that the subjects with rs4134816 CT or CC genotype had a decreased risk of NSCLC compared with those carrying TT genotype with OR (95 % CI) of 0.46 (0.26–0.84). We didn’t find that any other selected SNPs were associated with the risk of NSCLC.

**Table 3:** Genotype frequencies of *XAB2* and their association with non-small cell lung cancers

*XAB2* Genotypes	Controls (*n* = 470)		Cases (*n* = 470)		*OR* (95 % *CI*)^a^	*P* value^a^
	No	(%)	No	(%)		
rs4134816						
TT	429	91.3	450	95.7		
CT	40	8.5	20	4.3	0.49 (0.28–0.86)	0.012
CC	1	0.2	0	0.0	NC	
CT + CC	41	8.7	20	4.3	0.46 (0.26–0.84)	0.010
rs4134819						
AA	122	26.0	126	26.8		
AG	226	48.0	237	50.4	0.96 (0.70–1.31)	0.797
GG	122	26.0	107	22.8	0.81 (0.56–1.16)	0.251
rs794083						
CC	221	47.0	249	53.0		
CG	189	40.2	161	34.2	0.72 (0.54–0.96)	0.023
GG	60	12.8	60	12.8	0.87 (0.58–1.31)	0.517
rs4134860						
TT	341	72.6	332	70.7		
CT	118	25.1	120	25.5	1.01 (0.75–1.36)	0.962
CC	11	2.3	18	3.8	1.73 (0.80–3.75)	0.163
rs794078						
GG	365	77.7	374	79.6		
AG	93	19.8	94	20.0	0.97 (0.70–1.35)	0.871
AA	12	2.5	2	0.4	0.12 (0.03–0.54)	0.006
AG + AA	105	22.3	96	20.4	0.87 (0.64–1.20)	0.396

^a^Data were calculated by logistic regression and adjusted for sex, age (categories), and smoking status

### Stratification analysis of the *XAB2* polymorphisms and the risk of NSCLC

We then performed stratification analysis to evaluate the effect of environmental factors on the association of *XAB2* polymorphisms with the risk of NSCLC (Table 4). In dominant model, we found that the subjects carrying rs4134816 CC or CT genotype had a decreased risk for developing NSCLC among males with OR (95 % CI) of 0.39 (0.18–0.82), but not among females. When stratified by gender, we observed a positively significant interaction between rs4134816 genotypes and gender on decreasing NSCLC risk (*P* = 0.034). Our data also showed that younger subjects (age ≤ 60) with at least one C allele had a decreased risk of NSCLC with OR (95 % CI) of 0.35 (0.17–0.74), but older subjects didn’t. However, there was no gene-environment interaction observed (*P* = 0.094). We didn’t find that XAB2 4134816 C > T variant effect on the risk of NSCLC when stratified by smoking status. The environmental factors, such as age, sex and smoking had no effect on the risk of NSCLC related to XAB2 genotypes at other polymorphic sites (Table 4).

**Table 4:** Association of *XAB2* tagSNPs with NSCLC risk stratified by selected variables

Genetic Variant	Variable	Genotypes (Cases/Controls)		Dominant model (AB + BB)/AA^b^*OR* (95 % *CI*)^a^	*P* value
		AA^b^	AB + BB^b^		
rs4134816	Sex				
T > C	Male	314/301	10/27	0.39 (0.18–0.82)	0.013
	Female	136/128	10/14	0.68 (0.29–1.59)	0.370
	Age				
	≤60	251/254	10/29	0.35 (0.17–0.74)	0.006
	>60	199/175	10/12	0.98 (0.40–2.39)	0.970
	Smoking status				
	Non-smoker	252/256	13/24	0.51 (0.26–1.03)	0.060
	Ever-smoker	198/173	7/16	0.47 (0.19–1.20)	0.113
rs4134819	Sex				
A > G	Male	80/90	244/238	1.10 (0.77–1.58)	0.591
	Female	46/32	100/110	0.64 (0.38–1.09)	0.098
	Age				
	≤60	76/77	185/206	0.91 (0.63–1.33)	0.638
	>60	50/45	159/142	0.92 (0.57–1.48)	0.726
	Smoking status				
	Non-smoker	82/75	183/206	0.81 (0.56–1.17)	0.252
	Ever-smoker	44/47	161/142	1.13 (0.70–1.82)	0.628
rs794083	Sex				
C > G	Male	169/159	155/169	0.88 (0.64–1.20)	0.415
	Female	80/62	66/80	0.66 (0.41–1.05)	0.081
	Age				
	≤60	139/140	122/143	0.90 (0.64–1.27)	0.546
	>60	110/81	99/106	0.71 (0.47–1.07)	0.098
	Smoking status				
	Non-smoker	144/139	121/142	0.81 (0.58–1.14)	0.226
	Ever-smoker	105/82	100/107	0.74 (0.49–1.11)	0.141
rs4134860	Sex				
T > C	Male	227/237	97/91	1.14 (0.80–1.61)	0.470
	Female	105/104	41/38	1.11 (0.66–1.87)	0.702
	Age				
	≤60	186/211	75/72	1.24 (0.84–1.82)	0.275
	>60	146/130	63/57	0.97 (0.63–1.51)	0.906
	Smoking status				
	Non-smoker	190/207	75/74	1.10 (0.76–1.61)	0.608
	Ever-smoker	142/134	63/55	1.05 (0.67–1.63)	0.841
rs794078	Sex				
G > A	Male	256/256	68/72	0.92 (0.63–1.35)	0.673
	Female	118/109	28/33	0.79 (0.45–1.40)	0.426
	Age				
	≤60	208/226	53/57	1.04 (0.68–1.58)	0.872
	>60	166/139	43/48	0.74 (0.46–1.20)	0.226
	Smoking status				
	Non-smoker	211/225	54/56	1.02 (0.67–1.55)	0.930
	Ever-smoker	163/140	42/49	0.73 (0.45–1.18)	0.202

^a^Data were calculated by unconditional logistic regression and adjusted for gender, age (categories), and smoking status, where it was appropriate

^b^A stands for Major allele and B stands for Minor allele for each SNP

## Discussion

In this case-control study in a Chinese population, we found that two tag SNPs (rs794078 and rs4134816) in *XAB2* were associated with significantly decreased risk of development non-small cell lung cancer. These findings indicated that *XAB2* genetic variants might contribute to the susceptibility of lung cancer.

Nucleotide excision repair is the main mechanism for removing the bulky DNA adduct from damage DNA for preventing carcinogens-induced mutagenesis [22, 23]. Several animal models, where individual NER genes were disrupted, had showed the importance of the integrity of NER pathway in preventing lung cancer [24, 25].

TC-NER, as one of important sub-pathways in NER, only repairs the lesions in the transcribed strand in active genes. There are several major proteins involved in TC-NER in human cells, including CSA, CSB, XPA and XAB2. Studies have showed that the deficient of these nucleotide excision repair proteins contributed to the risk of various cancers. Animal experiments showed that the CSB played an important role in the cellular response to stress and CSB^−/−^ mice were increased susceptible to chemically induced skin cancer [26]. A case-control study also found 12.2 and 12.5 % reduced RNA transcriptional levels of CSA and CSB in lung cancer patients than controls [27].

*XAB2* is a key factor in TC-NER, which is composed of 855 amino acids and contains 15 tetratricopeptide repeat motifs. By interacting with CSA, CSB, RNA polymerase II and XPA, XAB2 conducted the multiple functions in the process of transcription and TC-NER [19, 20]. Microinjection of specific antibodies against XAB2 inhibits transcription and TC-NER, suggesting the key role of XAB2 in the process of transcription and TC-NER [20]. Knockdown of XAB2 in HeLa cell resulted in a hypersensitivity to killing by UV light and a decreased recovery of RNA synthesis [19]. Over expression of XAB2 was observed in HL60 cells treated with inhibited all-trans retinoic acid (ATRA) and inhibited XAB2 expression by small interfering RNA (siRNA) increased ATRA-sensitive cellular differentiation, which indicated that XAB2 was associated with the cellular differentiation [28].

Studies have demonstrated that the polymorphisms, which located in NER genes or regulatory sequences, may affect DNA repair capacity and further increase likelihood of cancer development. In the present study of NSCLC in Chinese, we used a relatively comprehensive selection of SNPs and found the significant effects of *XAB2* variants on the risk of lung cancer. This is the first study to investigate the association of *XAB2* polymorphisms with the risk for developing cancer. There were several studies to evaluate the role of *XAB2* genetic variants in complex autoimmune disease. For example, Briggs et al. conducted a case-control study to evaluate the correlation between *XAB2* rs4134860 T > C variant and the risk of multiple sclerosis (MS) and found an increased risk of MS among rs4134860 CC genotype carriers [29]. In this lung cancer case-control study, we didn’t find any association of *XAB2* rs4134860 T > C polymorphism with the risk of NSCLC. In another study, researchers analyzed the impact of several polymorphisms in DNA repair genes on the prognosis of colorectal cancer patients and didn’t find the association of *XAB2* rs794078 G > A variant with the cancer prognosis [30]. In present study, individuals carrying *XAB2* rs794078 AA genotype had 88 % decreased risk of NSCLC.

As we know, the magnitude of the effect of smoking far outweighed all other factors leading to lung cancer [31, 32]. Many studies have demonstrated that the strong association of smoking with lung cancer risk [5, 33, 34]. Therefore, we further analyzed the role of *XAB2* polymorphisms in the development of NSCLC stratified by smoking status. We observed that a 49 % protective effect for *XAB2* rs4134816 variant was evident only for non-smokers, but not for smokers. The exact mechanism of how cigarette-smoking effects on DNA repair capacity posted by *XAB2* polymorphism is unknown. One possible explanation may be that the protective effect of *XAB2* variant allele might be evident in non-smokers with low levels of oxidative damage. Similar pattern of genetic effects have been observed for DNA repair gene XRCC1 (X-ray repair cross-complementation group 1) at low smoking exposure, but not at high smoking exposure [35].

When stratified by gender, our study showed a 61 % protective effect of *XAB2* rs4134816 C genotype among men, but not among women. Genetic variants in NER genes are associated with variability of lung cancer risk. Letkova and his colleagues investigated the polymorphisms of selected DNA repair genes, including XPC, XPD, hOGG1 and XRCC1, and found the different risks of developing lung cancer when stratified by gender, which further supporting our current findings [36]. Our present study also found that a 65 % protective effect for *XAB2* rs4134816 T > C genetic variant among subjects aged 60 years or younger. Using Cox proportional hazard model, Gauderman et al. estimated the age-specific genetic incidence rate and found that the estimated proportion of lung cancer patients with high-risk allele exceeds 90 % for cases with onset at age 60 years or less and decreases to approximately 10 % for cases with onset at age 80 years or older. These findings suggested the contribution of age in the development of cancer [37]. The numbers of subjects in several of subgroups were very small, so some caution is needed when interpreting these findings.

Our study has its limitation. Due to the moderate sample size and the lack of related phenotypic and functional assays, large studies and functional evaluations are still need to be conducted in the future.

## Conclusions

In conclusion, we have genotyped 5 tag SNPs in *XAB2* gene in this NSCLC case-control set. We found the evidence of significant association with the risk of NSCLC for two tag SNPs (rs794078 and rs4134816) in *XAB2* gene in Chinese population. These results further supported that *XAB2* play a significant role in the development of NSCLC.

## Abbreviations

*XAB2*: XPA-binding protein 2
MAF: Minor allele frequency
OR: Odds ratio
CI: Confidence interval
SNP: Single nucleotide polymorphism
